# Supraceliac aortic cross-clamping to control bleeding from the celiac axis during pancreatic surgery: a case report

**DOI:** 10.1186/s40792-021-01343-z

**Published:** 2021-12-15

**Authors:** Yuki Takano, Shuichi Fujioka, Hironori Shozaki, Naoki Toya, Toru Ikegami

**Affiliations:** 1 Department of Surgery, Jikei University Kashiwa Hospital, 163-1 Kashiwa-shita, Kashiwa, Chiba, 277-0004 Japan; 2 Department of Hepatobiliary-Pancreatic Surgery, Jikei University School of Medicine, Tokyo, Japan

**Keywords:** Intraoperative injury, Celiac axis, Arterial clamping, Pancreatic surgery

## Abstract

**Background:**

Intraoperative bleeding from the celiac axis (CA) can occur during pancreatic surgery, and appropriate management is essential to avoid critical complications. Here, we have reported a case that was managed with supraceliac aortic cross-clamping (SAC) for arterial bleeding from the CA during pancreatic surgery.

**Case presentation:**

A 70-year-old man was diagnosed with pancreatic cancer located in the pancreatic head and body. Preoperative computed tomography showed a stricture at the root of the CA, which may have been caused by a median arcuate ligament. Pancreaticoduodenectomy with division of the median arcuate ligament was scheduled. Uncontrollable bleeding from the root of the CA was observed during surgery. The bleeding was controlled by performing SAC, and a defect in the CA was confirmed. Arterial wall repair was successfully performed under temporal blood control using SAC. The aortic clamp time was 2 min and 51 s, and the intraoperative blood loss was 480 ml.

**Conclusions:**

Although SAC is primarily a procedure for ruptured abdominal aortic aneurysm, it can be useful for the management of CA injuries during pancreatic surgery.

**Supplementary Information:**

The online version contains supplementary material available at 10.1186/s40792-021-01343-z.

## Background

The celiac axis (CA) is a major artery that is managed during pancreatic surgery, especially surgery for pancreatic cancer. Recently, distal pancreatectomy with CA resection has become a standard option for locally advanced pancreatic body/tail cancer with the development of neoadjuvant chemotherapy [1–3]. Therefore, intraoperative injury and uncontrollable bleeding from the CA are complications that can be encountered during pancreatic surgery. CA repair should be properly managed; otherwise, the patient may experience poor outcomes. Here, we have reported successfully management of CA injury through temporary aortic cross-clamping upstream of the CA during pancreatic surgery in a case.

## Case presentation

**Fig. 1:**
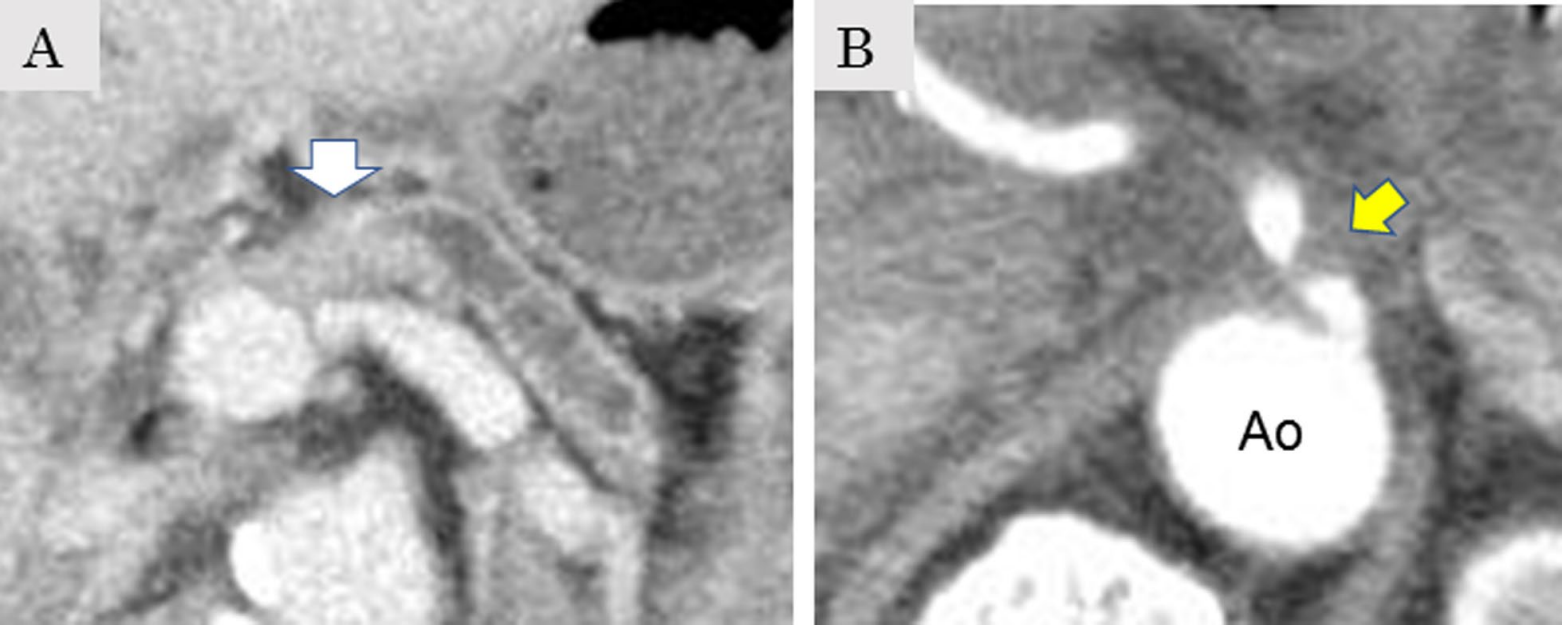
**A** Preoperative CT image which represents a resectable pancreatic cancer (white arrow). **B** Coincidental median arcuate ligament syndrome is shown (yellow arrow)

**Fig. 2:**
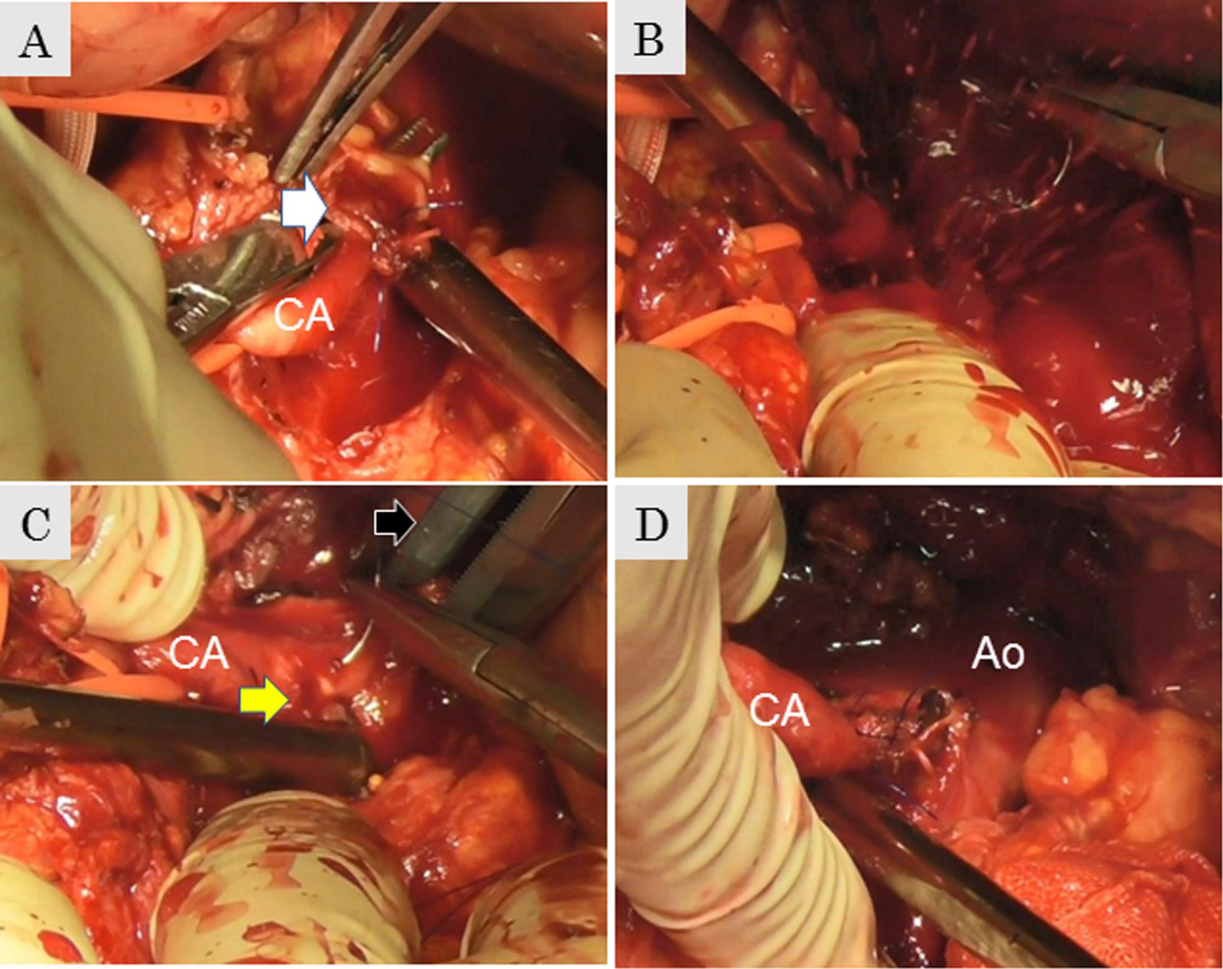
**A** Median arcuate ligament (white arrow). **B** Uncontrollable bleeding from the root of the CA. **C** A defect on the root of the CA (yellow arrow) recognized after performing supraceliac aortic cross-clamping (black arrow). **D** After the completion of CA repair, the clamp forceps are released. CA: celiac axis, Ao: aorta

A 70-year-old man was referred to our institution by his family doctor for the examination of repeated upper abdominal pain. Preoperative computed tomography (CT) suggested resectable pancreatic cancer of the pancreatic body (Fig. 1A) concomitant with stricture of the CA root (Fig. 1B), which may have been caused by median arcuate ligament (MAL). Pancreaticoduodenectomy with division of the MAL was scheduled. Unexpected bleeding around the CA was observed during surgery, which may have been caused by the injury incurred when the MAL was cut to release CA compression (Fig. 2A, B). As bleeding could be controlled by simple compression only, hemostasis by suturing was attempted first. Contrary to our expectations, the bleeding intensified, making it difficult to confirm the bleeding point. Therefore, we attempted supraceliac aortic cross-clamping (SAC) to manage bleeding. To expose the aorta, the crus of the diaphragm was divided, and the aorta was clamped upstream of the CA by a Fogarty vascular-clamp forceps. After performing SAC, the bleeding intensity significantly decreased and a defect of the adventitia measuring 7 mm in diameter on the CA was confirmed (Fig. 2C). The defect was repaired using a 4-0 Prolene continuous suture (Johnson & Johnson K.K, NJ, USA). The procedure time for SAC was 2 min and 51 s, and the intraoperative blood loss was 480 ml. The maximum blood pressure increased from 120 to 150 mmHg when SAC was performed and then decrease to 120 mmHg after releasing the clamp. The operative policy was changed to underdo distal pancreatectomy to decrease the risk of hepatic infarction. The patient was discharged uneventfully on postoperative day 19. A surgical procedure of SAC is shown in Additional file 1.

## Discussion

In our case, the unexpected bleeding may have been due to arteriosclerosis of the CA because fragility at the root of the CA was observed when the MAL was divided. Arteriosclerosis of the CA should be carefully evaluated by preoperative CT, because it can cause up to 60% stenosis of the CA [4]. In particular, the presence of irregular intraluminal shaggy changes of a certain length is a typical finding that suggests arteriosclerosis of the CA on the contrary compression due to the MAL [5]. In the present case, the stenosis at the root of the CA was diagnosed as compression due to MAL. Median arcuate ligament syndrome (MALS) is the condition which cause clinical presentation associated with direct compression of the celiac artery by the MAL [6]. As the MAL is originally fibrous attachment of the diaphragmatic crura, the treatment of the MALS is dividing the MAL. In most cases with pancreatic cancer, MAL is thought to be diagnosed incidentally by preoperative radiographic examinations, such as dynamic-CT or angiography. These “occult MALS” has considerable significance in performing pancreatic surgery, especially in pancreaticoduodenectomy because of the blockage of the bloodstream to the liver via the gastroduodenal artery. Under the circumstances of CA stricture due to arteriosclerosis, division of the median arcuate ligament has a risk of CA injury. In our case, we attempted SAC, which has been reported as a hemostatic technique for traumatic abdominal pathology [7]. Original procedure of the SAC has been described as exclusion of the aorta by incising the right crus through the lessor sac. This procedure can be easily performed during pancreatic surgery by dividing right crus cranially from the root of the CA. In the present case, the aorta was exposed by dividing the right crus with vessel-sealing device. When the SAC was performed, a defect in the adventitia of the root of the CA was recognized for the first time. This indicates that SAC is useful for temporalizing bleeding from major arterial injuries during pancreatic surgery.

Recently, endovascular vascular occlusion, such as resuscitative endovascular balloon occlusion of the aorta (REBOA), has attracted attention [8, 9]. For aortic injuries below the diaphragm, REBOA is an alternative to resuscitative thoracotomy (RT) with cross-clamping of the aorta to temporalize bleeding prior to cardiovascular collapse. In contrast, REBOA should be performed by an acute care surgeon or interventionalist trained in REBOA and a vascular surgeon for the management of possible vascular complications [10]. Therefore, SAC may be an option between RT and REBOA in the field of abdominal surgery. Easy access to the aorta upstream of the CA is a major advantage of SAC for pancreatic surgery, so as to take the supraceliac aorta immediately. In our case, it took only 3 min from the initiation of SAC to aortic cross-clamping. Thus, SAC is a useful option for the management of intraoperative arterial injuries during pancreatic surgery.

## Conclusion

Although SAC is primarily a procedure for ruptured abdominal aortic aneurysm, it can be useful for the management of CA injuries during pancreatic surgery.

## Supplementary Information

**Additional file 1.** Intraoperative management for injury of Celiac axis during pancreatic surgery.

## Data Availability

Data sharing is not applicable to this article as no datasets were generated or analyzed during the current study.

## Abbreviations

CA: Celiac axis
SAC: Supraceliac aortic cross-clamping
REBOA: Resuscitative endovascular balloon occlusion of the aorta
RT: Resuscitative thoracotomy
